# Contextual factors in maternal and newborn health evaluation: a protocol applied in Nigeria, India and Ethiopia

**DOI:** 10.1186/s12982-018-0071-0

**Published:** 2018-02-06

**Authors:** Kate Sabot, Tanya Marchant, Neil Spicer, Della Berhanu, Meenakshi Gautham, Nasir Umar, Joanna Schellenberg

**Affiliations:** 1 The Centre for Maternal, Adolescent, Reproductive and Child Health (MARCH), London School of Hygiene and Tropical Medicine, Keppel Street, London, WC1E 7HT UK; 2 Department of Disease Control, Faculty of Infectious and Tropical Diseases, London School of Hygiene and Tropical Medicine, Keppel Street, London, WC1E 7HT UK; 3 Department of Global Health, Faculty of Public Health and Policy, London School of Hygiene and Tropical Medicine, Keppel Street, London, WC1E 7HT UK

**Keywords:** Context, Contextual factors, Contextual moderators, Contextual indicators, External validity, Programme evaluation, Impact evaluation, Public health, Maternal health, Newborn health

## Abstract

**Background:**

Understanding the context of a health programme is important in interpreting evaluation findings and in considering the external validity for other settings. Public health researchers can be imprecise and inconsistent in their usage of the word “context” and its application to their work. This paper presents an approach to defining context, to capturing relevant contextual information and to using such information to help interpret findings from the perspective of a research group evaluating the effect of diverse innovations on coverage of evidence-based, life-saving interventions for maternal and newborn health in Ethiopia, Nigeria, and India.

**Methods:**

We define “context” as the background environment or setting of any program, and “contextual factors” as those elements of context that could affect implementation of a programme. Through a structured, consultative process, contextual factors were identified while trying to strike a balance between comprehensiveness and feasibility. Thematic areas included demographics and socio-economics, epidemiological profile, health systems and service uptake, infrastructure, education, environment, politics, policy and governance. We outline an approach for capturing and using contextual factors while maximizing use of existing data. Methods include desk reviews, secondary data extraction and key informant interviews. Outputs include databases of contextual factors and summaries of existing maternal and newborn health policies and their implementation. Use of contextual data will be qualitative in nature and may assist in interpreting findings in both quantitative and qualitative aspects of programme evaluation.

**Discussion:**

Applying this approach was more resource intensive than expected, in part because routinely available information was not consistently available across settings and more primary data collection was required than anticipated. Data was used only minimally, partly due to a lack of evaluation results that needed further explanation, but also because contextual data was not available for the precise units of analysis or time periods of interest. We would advise others to consider integrating contextual factors within other data collection activities, and to conduct regular reviews of maternal and newborn health policies. This approach and the learnings from its application could help inform the development of guidelines for the collection and use of contextual factors in public health evaluation.

**Electronic supplementary material:**

The online version of this article (10.1186/s12982-018-0071-0) contains supplementary material, which is available to authorized users.

## Background

Context is an important consideration when interpreting public health evaluation findings or contemplating implementation of a public health programme in a new setting. However, there are several challenges, among which is the imprecise and inconsistent usage of the word “context”.

Contextual factors, which we define as those elements of context that could affect implementation of a programme, can range from environmental disasters or political instability to health system weaknesses or the advent of another health programme. Collection and assessment of these contextual factors are critical to establishing internal and external validity of study findings. Those that are relevant to health programme evaluation are contextual factors that may confound or modify the effect of programmes on the outcome of interest, particularly for large scale programme evaluation where randomised studies are neither feasible nor appropriate [1]. In the absence of considering contextual factors, these insights and explanations for outcomes seen or unseen could be lost, leading to incorrect conclusions being drawn about the programme’s value. Furthermore there is a clear need to make evaluation findings useful not only to the programme in question, but also to provide information on transferability and applicability to inform decisions to scale up the programme or implement it elsewhere [2, 3].

There are several challenges faced by researchers incorporating context. The first is deciding which contextual factors are most relevant to capture. While all studies must be selective given the sheer magnitude of contextual factors that could be collected, one of the benefits of selecting a wide range of contextual factors is the ability to explore patterns in contextual factors that were not originally hypothesized to be directly relevant. Unfortunately, it’s often unclear what will be important until the analysis stage when changes that could be attributed to the intervention are revealed. This process is further complicated because of limited reliable secondary data sources at the desired unit of analysis and available during the appropriate time frame.

Public health practitioners can draw on academic disciplines and theoretical foundations to incorporate context, including: systems theory [4, 5], realist evaluation [6, 7], diffusion of innovations; [8, 9] normalisation process [10] policy analysis [11] and anthropology. Several evaluation frameworks also incorporate context [12–17]. These academic traditions and frameworks influence evaluator perspective on defining, collecting and using contextual information. For example, epidemiologists and more quantitatively orientated evaluators may view confounding and effect modification [see Table 1 for definitions] as the primary reason for collecting contextual factors and this affects the types of analyses they prioritise. Some quantitative researchers use randomisation as a way to deal with contextual variability, although there are limitations to randomised controlled trials and for complex or large scale evaluations this approach may be neither feasible nor appropriate, as mentioned above [18, 19]. Victora et al. [1] argue for contextual factors to be considered in randomised studies, as randomisation reduces but does not eliminate the risk of confounding, and randomised studies are often conducted in atypical conditions. This issue is particularly acute for cluster-randomised trials with a small number of clusters. Furthermore, randomisation does not address the issue of external validity. More qualitatively orientated researchers may argue that context is so deeply imbedded and intertwined with programmes that quantitative methods attempting to “control for context” are inherently invalid. Regardless of study design and paradigm, there is widespread agreement that evaluators should consider collecting contextual factors for use when interpreting their findings [20].

**Table 1:** Definitions of key epidemiological terms

*Internal validity* the extent to which the research tool is really measuring what it purports to measure [46]	
*External validity* the extent to which the research findings can be generalised to the wider population of interest and applied to different settings [46]	
*Confounding* a situation in which the estimate of association between an exposure and an outcome is distorted because of the association of the exposure with another factor that is also associated with the outcome. Confounding factors can be controlled for in certain analyses [47]	
Effect modification variation in the effect of the exposure on an outcome across values of another factor (effect modifier). Stratification allows for visualising the effect: rather than controlling for it, the effect of the exposure on the outcome would need to be reported separately for different values of the effect modifier [47]	

There are examples of complex public health evaluation studies that have incorporated contextual analysis to varying degrees [1, 5, 16, 21–32]. Few of these studies include details regarding how they selected contextual factors or how these data were collected and analysed, instead reserving limited word space in publications for other evaluation components. This lack of attention to contextual data reflects an emphasis on other evaluation priorities. A recent workshop of researchers who evaluate complex public health programmes discussed the extent to which context had featured in their past work and an overwhelming message from the proceedings was a call for renewed focus and emphasis on context [33].

Recently, the UK Medical Research Council (MRC) released guidelines for how to conduct process evaluations, expanding upon earlier guidelines for developing and evaluating complex health programmes that called for integrating process and outcome evaluation [34–36]. The new guideline defines context and process evaluation, highlighting their interrelated nature [see Table 2] [34]. This represents one of the most comprehensive efforts to date to set standards for how best to understand, measure and account for “context” in public health evaluation [35]. The MRC guideline embeds contextual analysis in process evaluation and represents a substantial movement forward in supporting a more consistent and transparent meaning of “context” and how it is used in public health evaluations. However specific guidance would be useful, including a minimum set of contextual factors to collect specific to areas of public health, how to assess data quality, how to analyse the data and how to use the findings.

**Table 2:** MRC guideline definitions of context and process evaluation

*Context* factors external to the intervention which may influence its implementation, or whether its mechanisms of impact act as intended [34]	
*Process evaluation* a study which aims to understand the functioning of an intervention, by examining implementation, mechanisms of impact, and contextual factors [34]	

In parallel, there have been efforts to make the process of adapting proven programmes to new contexts more rigorous. Gomm [37] a realist evaluator, developed a tool to analyse a programme’s context with specific vision for how to adapt to another context. Stirman et al. [38] developed a framework and coding system for documenting how programmes evolve during adaptation to new contexts. Bergstrom et al. [39] designed a tool to inform programme design in low and middle income countries that assesses organisational context through interviews with healthcare workers. Waters et al. [3] argue for the need to have a clear description of study context for the purposes of making systematic reviews more useful to decision makers. The Oxford Implementation Index aims to guide systematic review development through standardising review of implementation data, including which contextual factors should be collected in public health trials. The study of scale up and the role of contextual factors has been studied at length in the health policy field [40–43].

Most of these resources do not provide explicit guidance around which contextual factors are relevant for a maternal and newborn health programme evaluation. Bryce et al. [44] developed an evaluation framework for maternal, newborn and child health programmes that includes some detail about which contextual factors are relevant to collect. While this framework is the most specific, it leaves room for further recommendations around methods for collection, analysis and use.

Here we present an approach for the collection and use of contextual data in the evaluation of large scale complex maternal and newborn health (MNH) programmes in Ethiopia, Gombe state in northeast Nigeria and Uttar Pradesh in India [45]. We aimed to be consistent in how we defined context and prescriptive in how we captured relevant contextual information. In addition, we discuss how such contextual information has been applied to the interpretation of evaluation findings thus far and consequently the changes we have made to how we collect and use contextual information moving forward.

## Main text

### Methods/design

#### Process outline

**Fig. 1:**
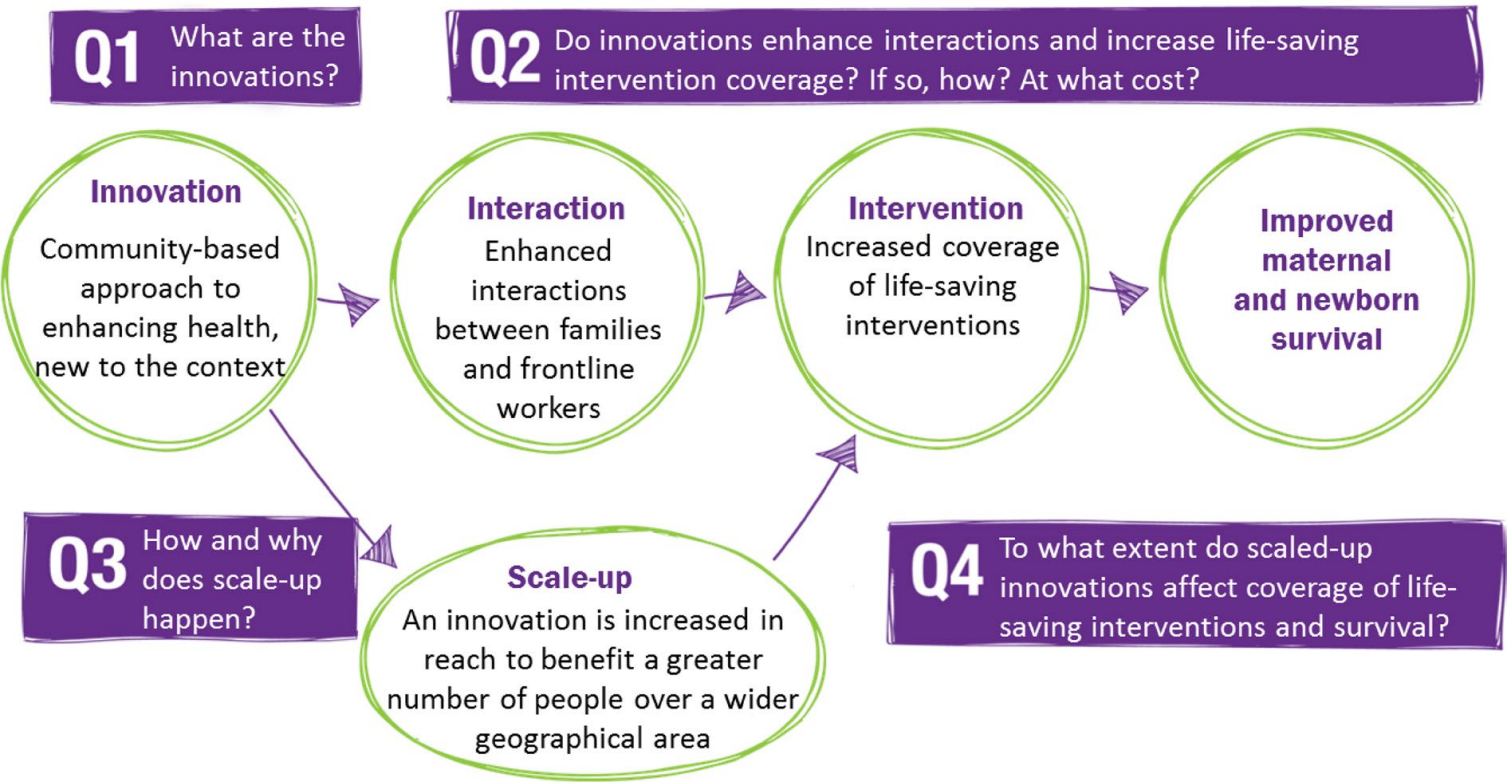
IDEAS learning questions. Visual of learning questions guiding broader research project

Informed Decisions for Actions to improve maternal and newborn health (IDEAS) developed research questions to test the Bill and Melinda Gates Foundation theory of change for their maternal, newborn and child health strategy [45]. These questions frame the domains of interest for contextual information and can be found in Fig. 1.

Activities designed to answer these questions include: before and after household and facility based surveys, in intervention and comparison areas, capturing interactions between families and frontline health workers and coverage of life saving interventions; community based qualitative inquiry on interactions between families and frontline workers; and qualitative interviews with a range of stakeholders on scale up. In each geography, we engaged local partners who managed data collection both for the evaluation studies and for contextual factors.

A scoping exercise was conducted in 2012 to assess feasibility of collecting and using contextual data, informed by the experience of researchers involved in similar evaluations [1, 32, 44]. It was immediately clear that the quantity, availability and quality of secondary data sources varied considerably between geographies and therefore we decided to take a more systematic approach that would include primary contextual data collection to fill gaps, the methods for which are described below.

We established an internal advisory group to oversee the implementation and focus on maintaining quality across the geographies. One staff member was assigned to work with each of the country-based teams collecting and reviewing these data to maintain consistency in implementation, document implementation and engage the advisory board as needed.

#### Defining context and selecting contextual factors

This section contains an overview of the methods: a list of the steps involved can be found in Table 3.

**Table 3:** Contextual data protocol process

Define context	
Determine list of relevant contextual themes and specific factors to obtain	
Categorise contextual factors to assign frequency of data needed for source to be appropriate	
Determine appropriate time frame for contextual data collection (period of time source documents collected). This should be aligned with programme implementation and timing of evaluation surveys. For example in an evaluation of a programme conducted from 2010 to 2012, baseline contextual factors should have been collected before 2010 and subsequent time periods ideally aligned with timing of evaluation surveys and/or following period of implementation	
Determine level of contextual factor aggregation most useful for evaluation (district, subnational, etc.)	
Share and adapt tools with country experts	
Conduct desk review to identify sources	
Extract data from sources	
Compile metadata on sources to understand frequency of availability, time frame of reference, geographic coverage and level of aggregation	
Iterative reviews of data	
Prepare maternal and newborn health policy summary to serve as baseline to assess policy changes over time	
Develop checklist for primary data collection	
Populate checklist with as much publicly available data as possible	
Circulate to research team to capture tacit knowledge	
Identify appropriate key informants with country specific experts and leads	
Conduct interviews using the populated checklist to verify existing information and fill gaps	
On an annual basis: update desk review and assess need for primary data collection	
Develop data analysis plan	
Integrate analysis into interpretations of study findings	

For the purposes of our work we defined context as the background environment, or setting for any programme. We defined contextual factors as those elements of context that could affect implementation of a programme. The list of contextual factors hypothesized to be relevant for evaluating coverage of evidence-based, life-saving interventions for maternal and newborn health fell into the following thematic areas:

- Demographics and socio-economics
- Epidemiological profile
- Health systems
- Health service uptake
- Infrastructure
- Education
- Environmental
- Politics, policy and governance.
- Maternal and Newborn health policy and implementation

Contextual factors were then split into two categories, with the intention of further focusing our time and effort by limiting data collection for category 1 relative to category 2 contextual factors. The categories are defined in the following way:

- *Category 1: Structural* Factors that were unlikely to change rapidly over the course of the evaluation. Examples include: religion or ethnicity of people living in a given geography. Leichter [11] refers to these as “slow changing” or “structural”.
- *Category 2: Situational* Factors hypothesized to change relatively quickly and thus require more frequent review, or as Leichter calls them, “situational,” that is, particular to a specific point in time [11]. Additionally, those of particular relevancy to understanding maternal and newborn health outcomes are also included in this category. Examples of this category include health programmes in the area, number of health care workers, vaccination campaigns, political instability or natural disasters.

The contextual factors were then classified as feasible to capture either through secondary data extraction from existing reports, or via primary data collection. Below, those two methods are described in greater detail. The intention was to repeat the secondary data extraction annually and repeat the primary data collection every 2 years as needed [see Tables 4 and 5 for the lists of contextual factors disaggregated by method of data collection].

**Table 4:** Contextual factors for secondary data extraction

Code	Contextual factor	Category 1 Structural 2 Situational
Demographic profile		
Dem 1	Total population	1
Dem 2	% Rural	1
Dem 3	% Urban	1
Dem 4	% Female	1
Dem 5	% Male	1
Dem 6	Population density (population/km^2^)	1
Dem 7	Fertility rate	2
Dem 8	Average family size	2
Dem 9	Religion	1
Dem 10	Ethnicity	1
Epidemiological profile		
Epi 1	Under-5-mortality rate	2
Epi 2	Maternal mortality rate	2
Epi 3	Newborn mortality rate	2
Epi 4	Infant mortality rate	2
Epi 5	Prevalence of malnutrition	2
Epi 6	% underweight	2
Epi 7	% stunting	2
Epi 8	% severe acute malnutrition	2
Epi 9	HIV-prevalence	1
Epi 10	Malaria transmission intensity	2
Health service provision		
HSP1	Number of family planning new users	2
HSP2	Number of family planning repeat users	2
HSP3	Number of women attending ANC (1st visit)	2
HSP4	Number of pregnant women attend 3 or more ANC visits	2
HSP5	Number of ANC clients receiving HIV test	2
HSP6	HIV-prevalence in pregnant women	2
HSP7	Number of pregnant women enrolled in HIV care	2
HSP8	Number of women delivering in a health facility	2
HSP9	Number of deliveries attended by skilled birth attendant	2
HSP10	Number of births protected against NNT	2
HSP11	Number of institutional maternal deaths	2
HSP12	Number of institutional neonatal deaths	2
HSP13	Number of first postnatal attendance	2
Health system		
HS 1	Number of hospitals	2
HS 2	Number of health centres (or equivalent)	2
HS 3	Number of health posts (or equivalent)	2
HS 4	Number of specialised doctor	2
HS 5	Number of general practioners	2
HS 6	Number of health officer	2
HS 7	Number of clinical nurse degree and diploma	2
HS 8	Number of midwife nurse degree and diploma	2
HS 9	Number of frontline health workers	2
HS 10	Number of rural frontline health workers	2
HS 11	Number of urban frontline health workers	2
HS 12	Number of ambulances available	2
HS 13	Overall health budget (allocated)	2
HS 14	Health budget per capita	2
HS 15	Available health budget (disbursed)	2
HS 16	% of budget allocated for maternal and child health	2
HS 17	% of population within 5 km of a health facility	2
Economics		
Eco 1	Ownership of assets-land/house	1
Eco 2	Employment rate	1
Eco 3	Coverage of electricity service	1
Eco 4	Wealth index (include definition)	1
Infrastructure		
Com 1	Mobile telephone coverage rate	1
Com 2	Mobile telephone subscriptions	1
Tran 1	Kilometers of all weather roads	1
Tran 2	% of local area connected to all weather roads	1
Wat 1	Proportion of population using improved drinking water source	2
Wat 2	Proportion of population using improved sanitation facilities	2
Education		
Ed 1	Number of primary schools	1
Ed 2	Number of secondary schools	1
Ed 3	Primary school net enrolment rate	1
Ed 4	% Male	1
Ed 5	% Female	1
Ed 6	Adult literacy rate	1
Ed 7	Female literacy rate	1
Environment		
Env 1	Average rainfall (annual in mm)	1
Env 2	Area affected by floods and rain (in hectares)	1
Env 3	Area affected by drought (in hectares)	1
Env 4	Total land mass (in hectares)	1

An excel table of the contextual factors identified for secondary data extraction, grouped into categories, coded and labelled as either structural or situational

**Table 5:** Primary data collection checklist

Contextual data checklist									Source of information
Code	Contextual factor	Local area 1							
		Circle yes/no	Circle coverage (language TBD) 1 = limited coverage 2 = 3 = 4 = 5 = all of local area						
Other health-related factors									
Have major health programmes, beyond those normally planned, been implemented in the following areas?									
OH 1	Malaria	Yes	No	1	2	3	4	5	
OH 2	Micronutrient supplementation	Yes	No	1	2	3	4	5	
OH 3	Nutrition	Yes	No	1	2	3	4	5	
OH 4	Immunization campaign	Yes	No	1	2	3	4	5	
OH 5	Other health programmes? Describe:	Yes	No	1	2	3	4	5	
Are the following NGOs active? (Ethiopia/Nigeria)/What are the most active NGOs? (India)									
OH 6	NGO 1	Yes	No	1	2	3	4	5	
OH 7	NGO 2	Yes	No	1	2	3	4	5	
OH 8	NGO 3	Yes	No	1	2	3	4	5	
OH 9	NGO 4	Yes	No	1	2	3	4	5	
OH 10	NGO 5	Yes	No	1	2	3	4	5	
OH 11	NGO 6	Yes	No	1	2	3	4	5	
Epidemiological									
OB 1	Have there been any major outbreaks? If yes, specify Disease, time period and proportion of area affected (coverage) for each outbreak below	Yes	No						
OB 2	Disease: time period (approx)			1	2	3	4	5	
OB 3	Disease: time period (approx)			1	2	3	4	5	
OB 4	Disease: time period (approx)			1	2	3	4	5	
Health system									
HSYS 1	Have there been any MNH policy changes since X policy (refer to desk review)?	Yes	No						
Have there been any stockouts of the following commodities (add timeframe)? What proportion of the local area was affected									
HSYS 2	Vaccines	Yes	No	1	2	3	4	5	
HSYS 3	Antibiotics	Yes	No	1	2	3	4	5	
HSYS 4	Medication (list to be specified)	Yes	No	1	2	3	4	5	
Infrastructure									
INF 1	Has there been construction of new roads?	Yes	No	1	2	3	4	5	
INF 2	Has there been construction of improved water supply?	Yes	No	1	2	3	4	5	
INF 3	Have sanitation facilities been improved?	Yes	No	1	2	3	4	5	
Environment									
DIS 1	Have there been any major environmental events (droughts/floods, etc.?)	Yes	No	1	2	3	4	5	
Political, policy and governance									
POL 1	Have there been any major political events?	Yes	No	1	2	3	4	5	
POL 2	Have there been any major government policy changes?	Yes	No	1	2	3	4	5	
Other contextual factors									
OTH 1	Is there anything else you would like to mention that could be influencing maternal and child health in X time period in these areas?	Yes	No						
Additional questions as needed									

An excel table of the contextual factors identified for primary data collection

#### Secondary data extraction

In partnership with collaborators based in Nigeria, Ethiopia and India, contextual factors from the multi-geography list were adapted and expanded to specific country contexts. At a minimum for each country the contextual factors were adjusted to reflect the relevant administrative areas and cadres of health care and frontline workers. Where available, data were disaggregated by age and gender.

A desk review was conducted within each country to identify availability of these contextual factors. Sources were identified, their frequency of data collection and coverage relative to our geographic areas of interest documented. Availability of sub-national data was noted as the programmes being evaluated were not implemented in all areas within these countries. Frequency of data collection was particularly relevant for Category 2 contextual factors as we will need to document change over the time period of programme implementation. Where multiple options for a given contextual factor existed, team members familiar with the geographies in conjunction with local partners assessed source quality and noted which source is more widely used.

The timing of data collection versus availability of reports was an anticipated challenge as ideally we would have sources collecting data around the same time as the evaluation survey data were collected. However even if the data were collected at the same time, reporting delays may impede data availability and therefore limit usability.

Following the desk review, the local partner extracted the data from these secondary sources for the time periods relevant to the evaluation studies (coinciding with baseline and midline surveys).

#### Policy summary

Following the desk review, secondary data extraction and prior to primary data collection, a maternal and newborn health policy summary was prepared by our local partners through document review of existing policies, strategies and programmes. The content of these policy memos included a brief introduction to each country including key demographic and maternal and newborn health statistics followed by a paragraph to half-page summary of each maternal and newborn health-related policy, strategy or programme. This draft was reviewed internally for comprehensiveness and quality. This served as the reference point for documenting implementation of existing policies and any policy changes captured through the primary data collection checklist.

#### Primary data collection

Primary data collection was supported by local partners. It began following the secondary data extraction and the preparation of the draft policy summary. The purpose of primary data collection was to:

1. Fill gaps in secondary data
2. Validate secondary data with advisory group and key informants and
3. Identify factors not originally hypothesized as being relevant
4. Identify changes related to maternal and newborn health policies and document implementation of existing maternal and newborn health policies

The finalised checklists (see Table 5) were populated with data from documents identified through the desk review and internet searches. The partially completed checklists were circulated to the advisory committee to ensure all internal tacit knowledge was maximised before approaching key informants.

Key informants were identified by in-country staff and senior staff of our local partner. The key informants were asked to review the checklist and provide information where gaps persist. This process started with centrally available individuals and snowballed to subnational levels. Approximately 5–10 key Informants (one or two per subnational geographic area) were anticipated to be involved per country. These structured interviews took place primarily in person. For example, in India, after informal, unstructured interviews with state-level government employees to help identify appropriate secondary sources, we interviewed district-level government employees including the district programme manager, assistant chief medical officer and chief medical officer.

Our approach combined secondary and primary data collection to address some of the limitations of existing data sources. In spite of these limitations we believed that the contextual data will lend insight into understanding public health programme implementation and are critically important to document and use in a standardised way as a best practice in public health programme evaluation.

#### Contextual data use

We outlined the following ways we intended to use this information in the testing of the specified theory of change:

- Interpret patterns in the quantitative and qualitative data used to evaluate if and how maternal and newborn health programmes increase coverage of life-saving interventions.
- Gather supplemental information to further understand explanations for how and why scale up happens and if these scaled programmes increase coverage of life-saving interventions
- Provide an opportunity through targeted interviews to ask questions of key informants about the preliminary findings to assist in interpretation.

Given reliance on secondary data sources, we anticipated some misaligned data collection timeframes and overall problems with quantitative data quality. Therefore, the specific uses of contextual data were always intended to be qualitative in nature.

Following the secondary data extraction and review of survey data for patterns in need of clarification, a more detailed data analysis plan was to be developed for each geography that would also identify existing information gaps to address during the primary data collection phase.

### Preliminary results

#### Implementation status

Implementation of this approach to collecting and using contextual factor data has been challenging. Each geography has had its own unique set of complications and solutions leading to variabilities across the geographies in how this approach was applied. Below is an illustrative example of our experience in Gombe, Nigeria.

In Gombe, there are few published reports available either at the federal level that reflect Gombe-specific data or within Gombe state. Limited data were extracted through in person interactions with government staff managing state-level databases and the rest was captured through expanded primary data collection. Costs were minimized through combining this with other data collection efforts for the MNH programme evaluation. After developing a first draft of the policy memo in Nigeria, we realized a key point of interest, the degree of policy awareness and implementation could only be ascertained through further primary data collection. Due to instability in Gombe, data collection to complete this part of the work was delayed.

The combination of limited secondary data availability and instability required adjustments to our approach and frequency of data collection. Based on our implementation experience in Gombe and elsewhere the following changes to the approach were made:

- *Policy memo development* was changed to become a two-staged process with a draft emerging following documentary review and a final version following primary data collection capturing policy awareness and implementation. This then further evolved from a one-off general maternal and newborn health policy memo to a more specific dashboard. The dashboard lists key maternal and newborn health care services and interventions and documents supportive policies and the degree of implementation for each country. Please see Additional file 1: Annex 1 for a list of the specific services and interventions screened for inclusion in existing policies and strategies.
- *Frequency of secondary data extraction* was changed to no longer be annual. Given the limited secondary sources in Nigeria and the delays in accessing data in the other geographies this frequency was impractical. Many of the data sources were only available in hard copy and some online platforms are not reliable. In the absence of updated central repositories it was not possible to implement this as envisioned.

Below are two illustrative examples of experiences using contextual data in Uttar Pradesh, India and Gombe State, Nigeria.

#### Uttar Pradesh, India

Researchers leading a facility readiness analysis conducted as part of the broader MNH evaluation suspected there was a supply chain variation between two types of facilities accounting for systematic differences in stocking of key MNH commodities. To test this hypothesis, we integrated a question related to this in our checklist. Ultimately the respondents were unaware of any differences in supply chains that could account for this distinction.

#### Gombe State, Nigeria

Results from the evaluation surveys in Gombe State, Nigeria showed an increase in syphilis screening at health facilities. Initially this was believed to be the result of the grantee innovations, the programmes we were evaluating. However, upon further investigation we learned that there was another project being implemented in these health facilities that focused on syphilis screening and that was likely to be the reason for the increase in syphilis testing. We learned of this program through informal means, as our formal contextual data collection activities were delayed in Nigeria. This experience highlights the importance of collecting and using contextual data—including informal information from colleagues in-country—for accurate interpretation of evaluation data.

These were the best examples of our approach to using contextual data.

#### Future plans

IDEAS was involved in the evaluation of community based newborn care (CBNC), which built upon components of Bill and Melinda Gates Foundation funded programs in Ethiopia and therefore represents an example of a scaled-up programme. The CBNC evaluation in Ethiopia integrated a limited set of contextual factors into the baseline and midline surveys. While this was not originally a part of our approach, this proved to be an easy and fruitful way to collect contextual data. One major advantage relative to the secondary contextual data extracted via the original approach is that the contextual factors collected in the surveys are explicitly linked to the relevant geographies and time periods of interest. Additionally, as the implementation of surveys requires authorization from woreda (district) leaders, this presents an opportunity to ask questions and collect primary data using the checklist. Across all three countries the content of the policy memos proved to be of particular interest to the funders of this research, the Bill and Melinda Gates Foundation. We envision that our approach will continue to evolve to maximise the efficiency of data collection and the value of the findings.

## Conclusions

Our intention in publishing this paper was to improve clarity on the potential use of contextual data and share with other researchers a set of steps for collecting and using contextual data for complex maternal and newborn health evaluation. The contextual factor data collected have thus far been less valuable than anticipated and we will continue to assess utility and adjust our approach as warranted.

Past experiences with capturing contextual data for the Integrated Management of Childhood Illness (IMCI) [1] and Expanded Quality Management Using Information Power (EQUIP) [32] reveal that substantial time and effort can be invested in collecting contextual data but not all of those efforts will yield meaningful information. While our implementation experience found that our approach needed to evolve, the learnings presented here could help inform the development of more specific guidelines for the use of contextual factors in public health evaluation with minimal resource use.

One of the major challenges this work faced was the decentralised nature of contextual factor data. National data repositories could ensure in-country ownership and improve use of such data. With the advent of the Sustainable Development Goals, national statistics offices may be resourced to meet the anticipated measurement mandate that extends well beyond the health sector. A knock-on effect of this would be higher quality, more easily accessible contextual data for public health evaluations.

In the meantime, in consultation with our government partners, we intend to make the data we have gathered publicly available in repositories, where feasible within the countries involved, for other health researchers who may have contextual factor needs that our efforts can address. While these contextual factors were selected with MNH programmes in mind, they would likely have applicability for other health programmes. In Ethiopia, work is underway to create a central data repository which may be an appropriate home for this data set. It is our hope that more countries will follow suit thus enabling greater local access to contextual factor data for MNH evaluations and beyond.

## Additional file

**Additional file 1: Annex 1.** Policy dashboard. An excel table of the services (proxies for access to care, facility readiness and interventions) screened for inclusion in existing policies, strategies and programmes. Includes: date of supportive policy, name of policy, relevant excerpt from policy and commentary on systems and implementation (if available).

## Abbreviations

CBNC: community based newborn care
EQUIP: Expanded Quality Management Using Information Power
IDEAS: Informed Decisions for Actions
MNH: maternal and newborn health
MRC: Medical Research Council
PROM: pre-term rupture of membranes
